# Refining cell-type annotation and fibrotic comparisons in chronic lung allograft dysfunction in single-cell RNA-seq studies

**DOI:** 10.1172/jci.insight.208925

**Published:** 2026-08-20

**Authors:** Allen Duong, Sajad Moshkelgosha, Tereza Martinu, Stephen Juvet

**Affiliations:** 1 Latner Thoracic Research Laboratories, Ajmera Transplant Centre, University Health Network, Toronto, Ontario, Canada.; 2 Institute of Medical Science, University of Toronto, Toronto, Ontario, Canada.; 3 Toronto Lung Transplant Program, Ajmera Transplant Centre, University Health Network, Toronto, Ontario, Canada.; 4 Division of Respirology, Department of Medicine, University of Toronto, Toronto, Ontario, Canada.

**Keywords:** Immunology, Pulmonology, Fibrosis, Macrophages, Transplantation

## Abstract

This commentary contextualizes recent findings in chronic lung transplant rejection, highlighting challenges in cell classification and the need for disease-specific fibrosis research.

**To the Editor:** We read with interest the recent article by Yan et al. (1), which identified, using single-cell transcriptomics, cell features and fibrotic signatures in chronic lung allograft dysfunction (CLAD). Important observations included the identification of cellular subsets enriched in CLAD (Fibro.AT2, Exhausted.T, and superactivated macrophages [Super.Macro]) as well as potentially targetable predicted interactions of KRT17^+^KRT5^–^ cells with myofibroblasts and macrophages. This study provides a strong comparison of CLAD with other pulmonary diseases such as idiopathic pulmonary fibrosis (IPF) and non-IPF interstitial lung disease. We would like to highlight two points that are important for interpreting these findings: the need for harmonized nomenclature when defining cell populations across studies and caution when extrapolating findings between CLAD and IPF given their distinct pathogeneses.

We were pleased to note the identification of Super.Macro, characterized by elevated expression of interferon-stimulated genes (ISG) and chemokines, as we previously identified these in both acute lung allograft dysfunction (ALAD) bronchoalveolar lavage (BAL) and bronchiolitis obliterans syndrome–type (BOS-type) CLAD lung tissue (2). These cells, which we called ISG macrophages based on prior literature (3), have a proinflammatory phenotype in vitro and have also been observed by others in ALAD BAL samples (4). To confirm that Super.Macro and ISG macrophages are in fact the same population, we performed an integration of the Yan et al. (1) and Khatri et al. (5) datasets with the Moshkelgosha et al. dataset (2). When subsetting and reclustering the monocyte/macrophage cell population, ISG macrophages were identified in ALAD BAL and within integrated CLAD lung samples (Figure 1A). The transcripts reported by Yan et al. in Super.Macro were assessed in BAL (Figure 1, B and D), confirming their identity with the ISG population. ISG macrophages were also observed in the integrated CLAD lung tissue dataset (Figure 1A) with expression of the same transcripts (Figure 1, C and E). Aside from lung transplantation, ISG macrophages have been documented in COVID-19 (6) and lung cancer (3), highlighting their role as a recurrent feature of proinflammatory pulmonary disease states associated with interferon signaling. As the field continues to evolve, we believe that harmonizing terminology across studies will be essential for enabling clearer interpretation and collective progress.

We also commend the authors for exploring aberrant KRT17^+^KRT5^–^ epithelial cells in CLAD with the use of spatial transcriptomics to identify fibrotic foci containing these cells colocalizing with myofibroblasts and macrophages. We note their use of IPF lung samples as a substitute for CLAD lungs owing to their shared fibrosis signaling pathways. However, we caution against drawing conclusions about CLAD pathogenesis from IPF tissue alone. There are essential differences between IPF and CLAD, suggesting distinct mechanisms, especially when considering the 2 major CLAD phenotypes: BOS and restrictive allograft syndrome (RAS). For example, BOS does not exhibit diffuse parenchymal fibrosis that is characteristic of IPF, whereas RAS does, albeit with a pattern distinct from usual interstitial pneumonia. The CLAD lungs reported in Yan et al. contain 7 BOS samples and 1 RAS sample, so it is possible that fibrotic pathways shared between IPF and CLAD are general features of fibrotic remodeling rather than disease or phenotype-specific mechanisms. An additional challenge is that many CLAD lungs contain mixed pathologies associated with both BOS and RAS phenotypes. In this context, spatial transcriptomic approaches may be particularly informative, as they could help distinguish molecular pathways associated with bronchiolar obliteration from those associated with parenchymal fibrosis and improve our understanding of mixed CLAD phenotypes. That said, the reported findings remain of interest, as the identification of shared fibrotic remodeling pathways and exploration of KRT17^+^KRT5^–^ fibrotic foci may inform the development of broadly applicable antifibrotic therapies. It would be of great value to assess differences between BOS and RAS-type CLAD, IPF, and other fibrotic lung diseases, especially when considering the added complexity of the alloimmune responses inherent in CLAD. The latter likely contribute to persistent injury and impaired healing, which result in a sustained fibroproliferative state.

In summary, Yan et al. offer valuable insights into the enriched cellular populations and fibrotic signaling in CLAD, especially given the scarcity and difficulty of acquiring CLAD lung samples (1). However, several challenges remain in the field. Differences in cell annotation across studies can complicate comparisons and limit the integration of findings across datasets. We therefore call for continued harmonization of cell population nomenclature and classification to reduce this complexity. In addition, while comparisons with IPF may provide useful context, the distinct pathogenesis of CLAD and the biological differences between BOS and RAS phenotypes underscore the need for disease-specific investigations. Future studies examining the differences between IPF and CLAD, as well as between BOS and RAS, are needed to better define disease-specific mechanisms of fibrosis. Together, these efforts will help refine our understanding of CLAD pathogenesis, improve cross-study interpretation, and support the development of effective interventions.

For detailed methods, information regarding sex as a biological variable, statistics, study approval, data availability, author contributions, and acknowledgments, see the supplemental materials (supplemental material available online with this article; https://doi.org/10.1172/jci.insight.208925DS1).

## Funding support

- Sanofi Innovation Awards (to SJ and TM).
- Cystic Fibrosis Foundation (JUVET18AB0 to SJ).
- Truscott-Parkes Catalyst Fund in Idiopathic Pulmonary Fibrosis (to SJ).
- Di Poce Research Scholar Award in Transplantation (to TM).

## Conflict of interest

The authors have declared that no conflict of interest exists.

## Supplementary Material

Supplemental data

## Figures and Tables

**Figure 1:**
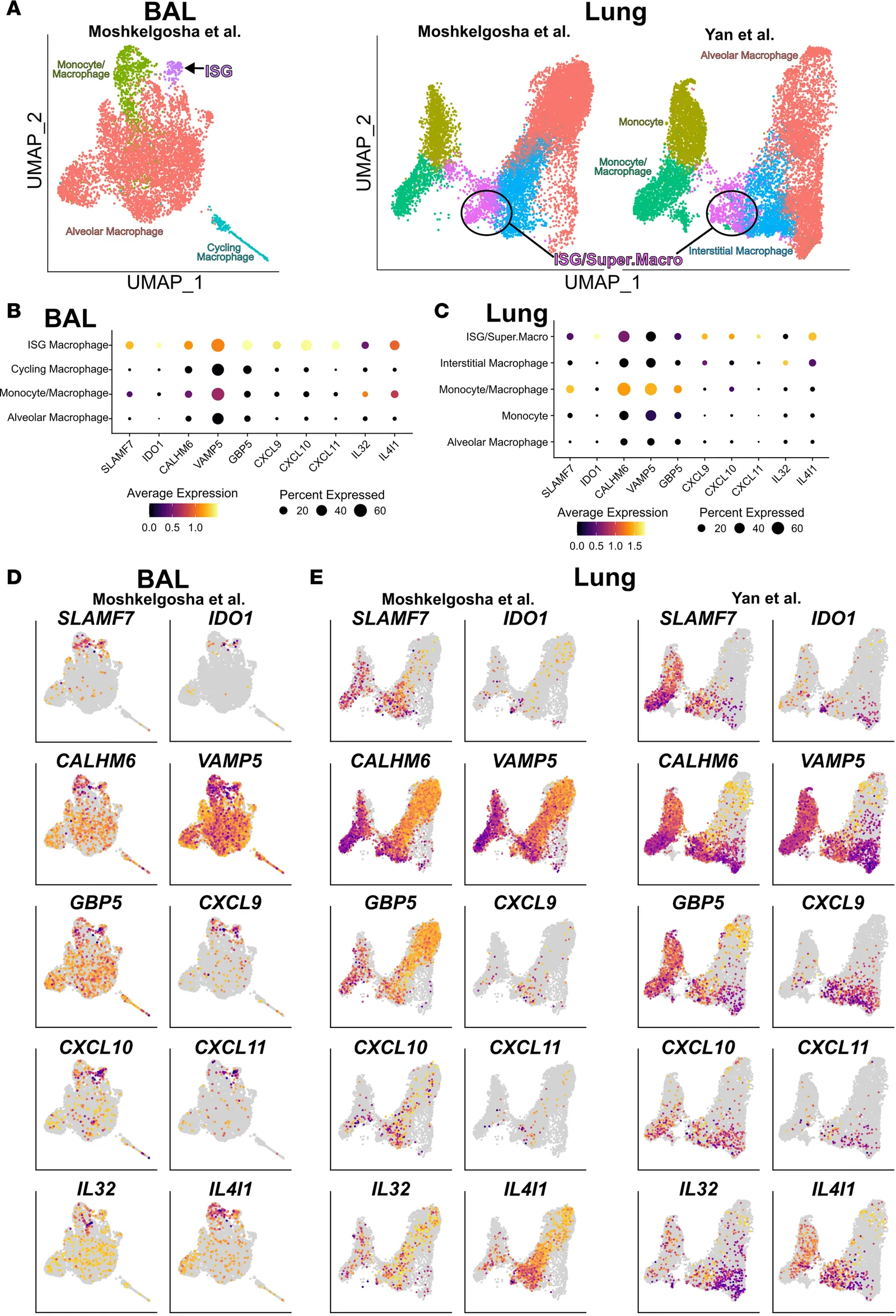
Expression of Super.Macro/ISG macrophage markers in ALAD BAL and CLAD lungs. (**A**) Uniform manifold approximation and projection (UMAP) plot displaying myeloid cells from ALAD BAL (left) and integrated CLAD lung (right) datasets, split by dataset and sample origin. (**B** and **C**) Dot plots showing the expression of Super.Macro/ISG macrophage-associated genes across monocyte/macrophage populations in BAL (**B**) and CLAD lung (**C**) datasets. Dot size represents the percentage of cells expressing each gene, and dot color represents the averaged scaled expression level. (**D** and **E**) Feature plots highlighting expression and distribution of Super.Macro/ISG macrophage-associated genes in BAL (**D**) and lung (**E**) datasets. Lung feature plots are displayed separately for Moshkelgosha et al. (2) and Yan et al. (1) cohorts.

## Version 1 08/20/2026

In-Press Preview

## Version 2 10/08/2026

Electronic publication
