# Supplementary material for: Refining cell-type annotation and fibrotic comparisons in chronic lung allograft dysfunction in single-cell RNA-seq studies
Source: JCI Insight. 2026 Aug 20;11(19):e208925. doi: 10.1172/jci.insight.208925 (PMC13726416; doi:10.1172/jci.insight.208925)
Supplement: Supplementary file 1 [file jciinsight-11-208925-s090.pdf]

Refining cell-type annotation and fibrotic comparisons in chronic lung allograft dysfunction in single-cell RNA sequencing studies

Allen Duong, Sajad Moshkelgosha, Tereza Martinu, Stephen Juvet

SUPPLEMENTAL MATERIAL

Methods

Sex as a biological variable.

Sex was not considered as a biological variable in this study.

Statistics.

Statistical analyses were performed using functions implemented in Seurat with default parameters. No additional statistical hypothesis testing was performed.

Reanalysis of published single-cell RNA sequencing datasets

Analysis was performed using Seurat package (v5.3.0) in R (v4.4.2). Individual datasets from Yan et al., Khatri et al. and Moshkelgosha et al., were downloaded from their respective repositories. Cells were filtered based on standard quality control criteria to exclude low-quality cells and potential doublets, retaining cells with 200 < nFeature_RNA < 5,000, 1,000 < nCount_RNA < 30,000, and mitochondrial transcript content < 15%. Data normalization, scaling and dimensionality reduction were conducted using default parameters in the Seurat package. Lung datasets were integrated using Harmony within Seurat, with principal component analysis as input to generate a corrected embedding. Uniform manifold approximation and projection (UMAP) were performed using the first 30 Harmony-corrected dimensions and clustering performed at 1.5 resolution. Resultant clusters were then annotated as alveolar macrophages, interstitial macrophages, cycling macrophages, monocyte/macrophages and pure monocyte populations based on differential gene expression of canonical marker genes. These marker genes include FABP4 and MARCO (for alveolar macrophages), LGMN and FOLR2 (for interstitial

macrophages), MKI67 and TOP2A (cycling macrophages), FCN1 and CD14 (monocytes/macrophages) and S100A8 and S100A9 (monocytes). Dot plots and feature plots were generated using Seurat and scCustomize (v3.2.0).

Study approval.

This study was approved by the University Health Network Research Ethics Board (protocols 15-9531, 11-0509, 11-0170).

Data availability.

All code used for data processing and analysis is available at https://github.com/Allen-Duong-uhn/CLAD-scRNAseq-crossstudy-analysis/.

Author Contributions.

A.D. contributed to data acquisition, formal analysis, data visualization, and preparation of the original draft of the manuscript. S.M. contributed to data acquisition, methodology development and formal analysis. T.M. and S.J. contributed funding, resources, and supervision. All authors contributed to the review and editing of the manuscript and approved the final version.

Acknowledgements.

The authors thank the investigators of the original studies for making their single-cell RNA sequencing datasets publicly available.
